# The emerging role of estrogen's non-nuclear signaling in the cardiovascular disease

**DOI:** 10.3389/fcvm.2023.1127340

**Published:** 2023-04-12

**Authors:** Hiroyuki Tokiwa, Kazutaka Ueda, Eiki Takimoto

**Affiliations:** ^1^ Department of Cardiovascular Medicine, Graduate School of Medicine, The University of Tokyo, Tokyo, Japan; ^2^ Division of Cardiology, Department of Medicine, Johns Hopkins University School of Medicine, Baltimore, MD, United States

**Keywords:** estrogen, non-nuclear signaling, cardiovascular disease, genetically modified animal, membrane-initiated steroid signaling

## Abstract

Sexual dimorphism exists in the epidemiology of cardiovascular disease (CVD), which indicates the involvement of sexual hormones in the pathophysiology of CVD. In particular, ample evidence has demonstrated estrogen's protective effect on the cardiovascular system. While estrogen receptors, bound to estrogen, act as a transcription factor which regulates gene expressions by binding to the specific DNA sequence, a subpopulation of estrogen receptors localized at the plasma membrane induces activation of intracellular signaling, called “non-nuclear signaling” or “membrane-initiated steroid signaling of estrogen”. Although the precise molecular mechanism of non-nuclear signaling as well as its physiological impact was unclear for a long time, recent development of genetically modified animal models and pathway-selective estrogen receptor stimulant bring new insights into this pathway. We review the published experimental studies on non-nuclear signaling of estrogen, and summarize its role in cardiovascular system, especially focusing on: (1) the molecular mechanism of non-nuclear signaling; (2) the design of genetically modified animals and pathway-selective stimulant of estrogen receptor.

## 1. Introduction

Cardiovascular disease (CVD) is the leading cause of death in many countries, and its total burden is increasing dramatically (1–3). Sexual dimorphism has been observed in various CVDs. Women are less susceptible to coronary artery disease than men; however, their morbidity increases after menopause, reaching male levels (4, 5). Furthermore, menopause is associated with an increased prevalence of metabolic syndrome, a CVD risk factor (6, 7). These findings suggest a cardiovascular protective role of female sex hormones, especially estrogen, which have been consistently reported in basic research. Although hormone replacement therapy (HRT) was expected to decrease CVDs in postmenopausal women, a randomized controlled trial (RCT) by the Women's Health Initiative failed to demonstrate an improvement in CVD morbidity and was terminated early owing to adverse events including breast cancer (8). However, subanalysis of the RCT revealed conjugated equine estrogens had a tendency to lower CVD morbidity in relatively early postmenopausal women (9). Additionally, oral estradiol administration suppressed carotid artery atherosclerosis when treatment was initiated within six years, but not ten or more years after menopause (10). These reports indicate that HRT can induce cardiovascular benefits with careful application and encourage further research on the molecular mechanisms of estrogen signaling.

Estrogen receptors (ERs) regulate gene expression as transcription factors in the nucleus, known as nuclear signaling. However, a subpopulation of ERs is present at the plasma membrane and initiates intracellular signaling, referred to as “non-nuclear signaling” or “membrane-initiated steroid signaling”. Despite their relatively small numbers compared to nuclear ERs (11, 12), an increasing body of evidence suggests the essential role of non-nuclear signaling in various physiological functions, including cardiovascular effects (13, 14). Additionally, G-protein-coupled estrogen receptor (GPER), a distinct subtype of ER, has been identified as another mediator of non-nuclear signaling.

In this article, we first describe the characteristics of ERs and the molecular mechanism of non-nuclear signaling. Next, the role of non-nuclear signaling in cardiovascular systems is discussed through studies using genetically modified animals and pathway-selective stimulators. A concise review of GPER is also provided.

## 2. Structure and ligand of estrogen receptors

Endogenous estrogens exert physiological effects by binding to their receptors (ERs). Two subtypes of ERs, ER*α* and ER*β*, belong to the nuclear hormone receptor superfamily and share common structural characteristics (15–17). ER consists of six distinct domains (A to F domains) (18). The N-terminal A/B domains contain a transcriptional activation domain (AF1), which facilitates the transcriptional function of ER. The C domain is a DNA-binding domain (DBD) that interacts with a specific DNA sequence called estrogen response elements (EREs) located in the transcriptional regulatory region of estrogen-responsive genes. The D domain is a flexible hinge region between domains C and E, and contains a nuclear localization signal (NLS) and a nuclear export signal (NES). The E domain corresponds to the ligand-binding domain (LBD), which harbors another transcriptional activation domain (AF2). The C-terminal of ER is the F domain. Although ER*α* and ER*β* are encoded by two genes, their C and E domains are highly homologous (19), while the other domains are relatively divergent (20). In addition, splicing variants of ER*α* and ER*β* have distinct physiological functions (18).

Upon binding to their ligands, ERs undergo a conformational change and form a stable dimer (21–23), which then enter the nucleus guided by NLS (24–26). ERs regulate gene expression with associated coregulators (27–30), and phosphorylation of ER also enhances their transcriptional activity in a ligand-independent manner (31–33).

Estradiol (E2) is the most potent endogenous estrogen in pre-menopause women, whereas estrone (E1) plays a larger role after menopause, and estriol (E3) shows a greater importance during pregnancy (34). Estetrol (E4) is synthesized during pregnancy by fetal liver enzymes (35). Additionally, various natural and synthetic exogenous compounds act as ER ligands (36). A group of synthesized estrogenic compounds, known as selective estrogen receptor modulators (SERMs), exhibit dual functionality as both agonist and antagonist of ER in different organs due to tissue- or cell-specific difference in the recruitment of cofactors (36, 37). It has been reported that E4 exhibits the activity of an natural SERM (38).

Some oxysterols, which are oxygenated derivatives of cholesterol, function as ER ligands. 27-hydroxycholesterol (27HC) inhibits E2-induced nitric oxide synthase expression and re-endothelialization of murine carotid artery (39). In contrast, 27HC promotes breast cancer progression in an ER-dependent manner (40, 41), suggesting its characteristic as an endogenous SERM (42). 27HC also regulates bone homeostasis, partially mediated by ERs (43, 44). Similarly, 25-hydroxycholesterol exhibits ER*α*-mediated breast and ovarian cancer cell proliferation and prevents hypoxia-induced cardiomyocyte apoptosis (45).

## 3. Non-nuclear signaling of ERs

### 3.1. Mechanism of plasma membrane localization

In addition to their role in nuclear signaling, ERs also mediate rapid intracellular signaling. In 1967, Szego and Davis showed that estrogen increased cyclic adenosine monophosphate (cAMP) concentration in the rat uterus within minutes (46). Following studies documented rapid calcium uptake of endometrial cells after E2 administration and E2 binding to the cell membrane (47, 48). Further research has identified the existence of membrane-bound ER*α* and ER*β* (11, 49, 50), which are responsible for rapid signaling, such as the activation of extracellular signal-regulated kinase (ERK), protein kinase B (PKB, also known as Akt), and endothelial nitric oxide synthase (eNOS) (51–54). This signaling is referred to as “non-nuclear signaling” or “membrane-initiated steroid signaling”. However, it should be noted that this signaling can also induces the transcriptional response subsequently (55, 56).

Palmitoylation, a posttranslational modification of ER plays an essential role in trafficking to the plasma membrane (57–60). A conserved amino acid motif in the E domain of ER*α* and ER*β* is responsible for palmitoylation (61) by DHHC-7 and −21 (62). Caveolin-1, the main component of caveolae (63), is colocalized with ER*α* (64) and the amino acid substitution of S522A in ER*α* impairs the interaction with caveolin-1 and plasma membrane localization (65). Striatin, a scaffold protein, is another component of the signaling complex of membrane-bound ER*α* (66).

### 3.2. Signaling complex of membrane-localized ER

**Figure 1:**
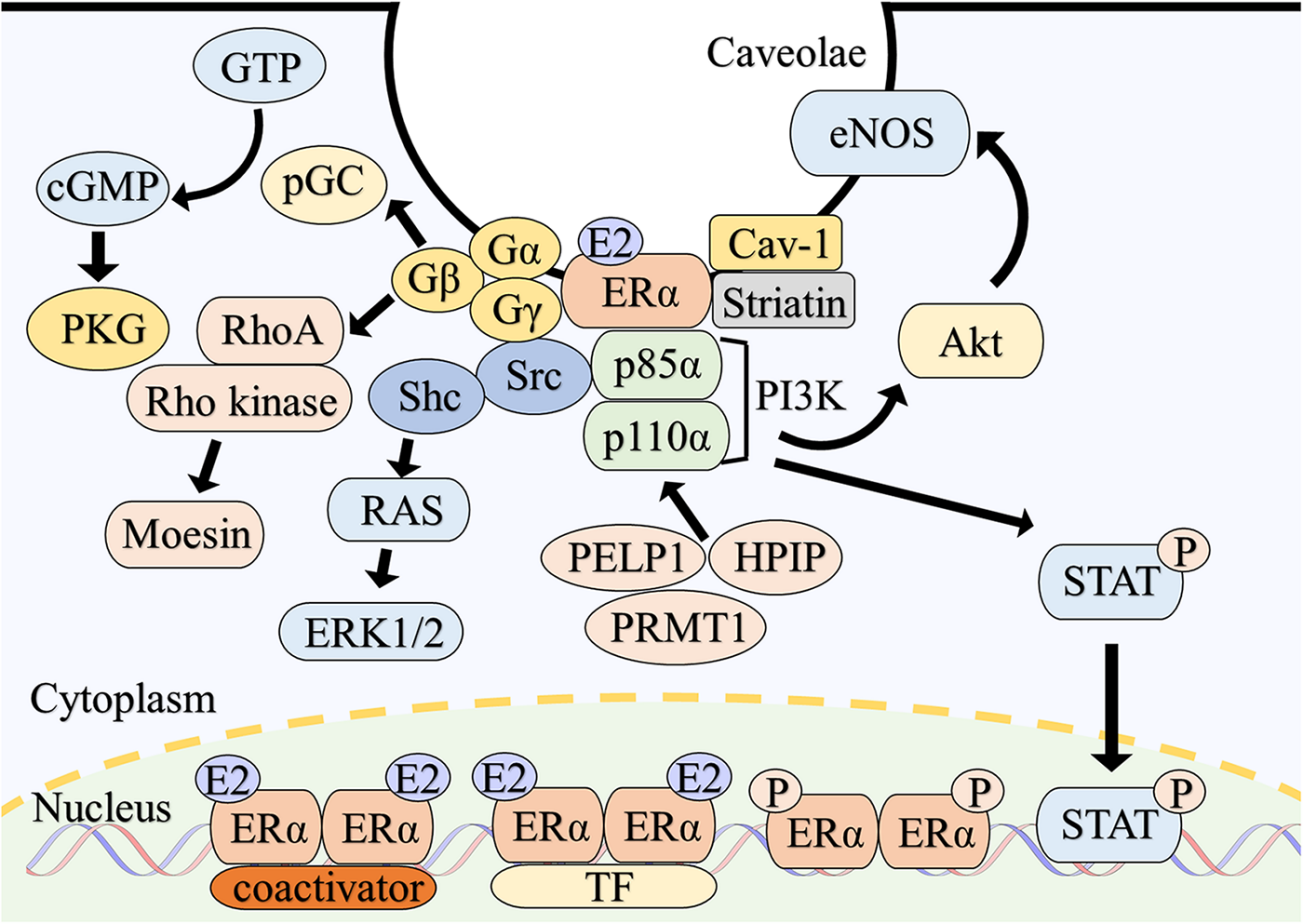
Overview of intracellular signaling of estrogen receptor *α*. Classically, E2-bound ER*α* dimerizes and translocate to the nucleus. ER*α* directly binds to estrogen response elements of the target genes with coactivators and modulates gene expressions. ER*α* also binds to DNA indirectly in association with other transcription factors. Phosphorylation of ER*α* also enhances transcriptional activity. A subpopulation of ER*α* is localized to the caveolae of the plasma membrane through the interaction with caveolin-1 and striatin. ER*α* on the plasma membrane assembles a functional complex with associated proteins such as G proteins, Src and PI3K, resulting in the rapid activation of multiple intracellular signaling. Abbreviations; Akt, protein kinase B; Cav-1, caveolin-1; cGMP, cyclic guanosine monophosphate; eNOS, endothelial nitric oxide synthase; E2, estradiol; ERK, extracellular signal-regulated kinase; ER*α*, estrogen receptor *α*; GTP, guanosine triphosphate; HPIP, hematopoietic PBX-interacting protein; PELP1, proline-, glutamic acid- and leucine-rich protein 1; pGC, particulate guanylate cyclase; PI3K, phosphatidylinositol-3 kinase; PKG, cGMP-dependent protein kinase; PRMT1, protein arginine methyltransferase 1; P, phosphorylation; Shc, src homology and collagen; STAT, signal transducer and activator of transcription; TF, transcription factor.

On the plasma membrane, ERs form functional modules with associated proteins (Figure 1), including G-protein. In human umbilical vein endothelial cells, membrane ER*α* interacts with G*α*_13_, which activates the RhoA/Rho Kinase/Moesin pathway and induces cell migration (67). Furthermore, E2-bound membrane ER*α* links to G*α*i-2/3 and stimulate particulate guanylate cyclase-A, which causes the generation of cyclic guanosine monophosphate (cGMP). In consequence, activated cGMP-dependent protein kinase (PKG)-I stimulates cystathionine *γ*-lyase in endothelial cells, resulting the rapid release of hydrogen sulfide, which acts as a vasodilator (68). Human ER*α* binds to G*α*i and G*βγ* at amino acids of 251–260 and 271–595 of ER*α* respectively. Disruption of the interaction of ER*α* with G*α*i or G*βγ* inhibits E2-induced Src and ERK phosphorylation (69). Point mutations in the G*α*i-binding domain of ER*α* diminish E2-stimulated activation of ERK and eNOS (70).

Src, a proto-oncogene, plays a critical role in Ras/ERK activation by E2-bound ER*α* (71). Src phosphorylates human ER*α* at Tyrosine 537 (72), and the SH2 domain of Src subsequently binds to ER*α*, modulating Src activity (73). A similar mechanism is observed with ER*β* (73). Disruption of the ER*α*/Src association inhibits E2-induced proliferation of MCF-7 cell, which is an estrogen-responsive tumor cell (74). Src homology and collagen (Shc) is also contribute to ERK1/2 activation, with Src acting as an upstream regulator of Shc (75). Additionally, Proline-, glutamic acid- and leucine-rich protein 1 and hematopoietic PBX-interacting protein assist in the complex formation of ER*α*, Src and p85*α* subunit of phosphatidylinositol-3-OH kinase (PI3K) (76, 77). Protein arginine methyltransferase 1 is also involved in this process, mediating the methylation of ER*α* arginine 260 (78).

Nitric oxide production by eNOS is a vital function of endothelial cells (79). E2-bound ER*α* on the plasma membrane binds to the p85*α* subunit of PI3K and rapidly stimulates eNOS *via* the PI3K-Akt pathway (52, 80), primarily in caveolae (81). G*α*i and heat shock protein 90 are also involved in E2-induced eNOS activation (82, 83).

While studies on non-nuclear ER*β* signaling are limited, it has been reported that ER*β* activates eNOS in endothelial cell caveolae (84). Additionally, both ER*α* and ER*β* activate ERK1/2 and Akt in a subtype-specific manner (85).

## 4. Genetically modified animal models for ER non-nuclear signaling

Several genetically modified mouse models have been generated to investigate the role of non-nuclear signaling of ER*α* function (Table 1). Although the specific method of inhibiting non-nuclear signaling differ in each mouse model, these mice consistently exhibit a lack of rapid activation of eNOS by E2, which is known to play a pleiotropic role in maintaining cardiovascular homeostasis (107).

**Table 1:** Comparison of the phenotypes of genetically modified mouse models.

	C451A-ER*α*	R264A-ER*α*	DPM	KRRKI	ERα^KI/KI^Tie2^Cre^	ERαAF1^0^	ERαAF2^0^	H2NESKI	GPER knockout
Female fertility	Infertile (86)	Fertile (87)	Fertile (88)	Infertile (89)	Fertile (90)	Infertile (91)	Infertile (92)	Infertile (93)	Fertile (94)
Uterine morphology	Atrophic (86)/Normal (95)	Normal (87)	ND	Normal (89)	Normal (90)	Atrophic (96)	Atrophic (96)	Atrophic (93)	Normal (94)
Uterine hypertrophy by E2	Impaired (86)/Preserved (95)	Preserved (87)	ND	Preserved (89)	Preserved (90)	Impaired (91)	Impaired (92)	Impaired (93)	Preserved (94)
Vascular effect of E2									
Acceleration of reendothelialization	Abrogated (95)	Abrogated (87)	ND	ND	Abrogated (90)	Preserved (91)	Preserved (92, 95)	Augmented without E2 (93)	ND
Inhibition of neointimal hyperplasia (mechanical wire injury model)	ND	ND	Abrogated (88)	ND	Abrogated (90)	Abrogated (97)	ND	ND	ND
Prevention of atherosclerosis	Preserved (98)	Preserved (87)	ND	ND	ND	Preserved (91)	Abrogated (92, 98)	ND (no statistically significant change) (93)	Abrogated (99)
Cardiac phenotype	ND	ND	ND	Impairment of E2-dependent cardioprotection by PDE5 inhibitor (89)	ND	ND	ND	ND	Impairment of E2 protection against ischemia/reperfusion injury (100) Systolic and diastolic dysfunction (cs-GPER KO) (101, 102)
**Metabolic disorders**									
Body weight	No effect (103)	ND	ND	Increased (104)	ND	No effect (96)	Increased (96)	Increased (93)	Increased (105) /Decreased (106)
Visceral fat accumulation	No effect (103)	ND	ND	Increased (104)	ND	No effect (96)	Increased (96)	Increased (93)	Increased (105)
Glucose intolerance	Mildly impaired (103)	ND	ND	Impaired (104)	ND	Mildly impaired (96)	Impaired (96)	Impaired (93)	Impaired (106)

ND, not determined; cs-GPER KO, cardiomyocyte-specific GPER knockout.

The C451A-ER*α* mouse model, which is characterized by the inhibition of palmitoylation and translocation of ER*α* to the plasma membrane by the substitution of cysteine 451 with alanine, exhibits a complete absence of membrane-localized ER*α* (86, 95), leading to the abrogation of non-nuclear signaling.

Based on the importance of human ER*α* amino acids 251–260 in non-nuclear signaling (69, 70), the R264A-ER*α* mouse model was generated by replacing arginine 264 of murine ER*α* with alanine, which corresponds to arginine 260 of human ER*α* (87). While the C451A-ER*α* female mouse exhibits infertility and impaired reproductive organ development (86), the R264A-ER*α* female mice remain fertile with intact reproductive organs, which suggests a difference in the degree of non-nuclear signaling inhibition between the two models.

Another mouse model of non-nuclear signaling inhibition was produced by disrupting the interaction between ER*α* and striatin, which is facilitated by amino acids 183–253 of human ER*α* and is crucial for the membrane localization of ER*α* (66). The disrupting peptide mouse (DPM) was generated through transgenic overexpression of a peptide containing the amino acid sequence of ER*α* 176–253, which disrupts the interaction between ER*α* and striatin. DPM mice exhibit a lack of rapid E2-induced phosphorylation of Akt or ERK in endothelial cells, resulting in the failure to activate eNOS (88).

By developing the concept of DPM mice, it was discovered the substitution of lysine 231, arginine 233 and 234 into alanine (KRR to AAA) of human ER*α* inhibits its interaction with striatin (108). In a human endothelial cell line with modified ER*α*, the rapid activation of ERK, Akt, and eNOS by E2 administration is abrogated, while the direct genomic reaction is preserved. To investigate the effects of this modification *in vivo*, a mouse model was established in which the endogenous ER*α* was replaced with the modified ER*α* (KRR knock-in: KRRKI mice) (104).

Recently, a novel mouse model called ER*α*^KI/KI^Tie2^Cre^ mice was established. In this mouse model, ER*α* non-nuclear signaling is inactivated by disrupting its binding to the p85*α* subunit of PI3K through an arginine 263 to alanine mutation in a tissue-specific manner under the Cre-loxP system (90).

In this section, we summarize the findings obtained from these models and compare them with the mouse model with inactivated nuclear signaling, specifically with regards to the cardiovascular and metabolic systems.

### 4.1. Protection against vascular injury

Estrogen accelerates re-endothelialization and suppresses neointimal hyperplasia after vascular injury, with ER*α* playing an essential role (109, 110). Neither the C451A-ER*α*, R264A-ER*α* nor ER*α*^KI/KI^Tie2^Cre^ mice exhibits E2-induced acceleration of re-endothelialization after electric perivascular injury (87, 90, 95). Similarly, E2 administration does not improve neointimal hyperplasia after mechanical wire injury in DPM or ER*α*^KI/KI^Tie2^Cre^ mice (88, 90). In contrast, the mouse models with inactivated nuclear signaling by ER*α* AF1 or AF2 domain deletion (ER*α*AF1^0^, ER*α*AF2^0^) have demonstrated preserved E2 acceleration of carotid artery re-endothelialization after electric injury (91, 92, 95).

These findings suggest that ER*α* non-nuclear signaling plays the predominant role in the vascular protection by estrogen. This idea is supported by previous research demonstrating that E2 suppresses the proliferation of vascular smooth muscle cells (VSMCs), an underlying mechanism of neointimal hyperplasia (111). This effect is mediated through the formation of a complex of membrane ER*α* and striatin and protein phosphatase 2A (PP2A), leading to subsequent kinase inactivation (112). In VSMCs derived from DPM mice, the estrogen-induced complex formation and anti-proliferative effect is abrogated.

However, it is worth noting that E2-induced suppression of neointimal hyperplasia after mechanical wire injury is abolished in ER*α*AF1^0^ mice (97). This result suggests that ER*α* nuclear signaling may also contribute to vascular protection or that there may be differences in the underlying biological mechanism of each vascular injury model.

The mouse models with genetic modification within D-domain provide valuable insight into the function of non-nuclear signaling in vascular protection. Amino acid substitution in the hinge region and NES of ER*α* D-domain alter the pattern of intracellular distribution of ER*α*. While wildtype ER*α* predominantly is localized in the nucleus, ER*α* with modifications in the putative NLS of the hinge region and NES (H2 + NES ER*α*) is exclusively localized in the cytoplasm. This altered localization is assumed to be caused by enhanced NES function, which is partially restored by leptomycin B, a nuclear export inhibitor. H2 + NES ER*α* exhibits impaired transcriptional activity but maintains a non-nuclear response of ERK1/2 phosphorylation after E2 administration (113, 114). The mouse model with mutated ER*α* (H2NESKI) exhibits an interesting cardiovascular phenotype where the degree of carotid artery re-endothelialization after electric injury is similar to that of E2-treated wild-type female mice, even in the absence of estrogen by ovariectomy. This observation suggests an intrinsically enhanced non-nuclear ER*α* signaling in H2NESKI mice (93).

### 4.2. Atherosclerosis prevention

Estrogen reduces the development of atherosclerotic lesion through ER*α* in mouse models of atherosclerosis with apolipoprotein E-deficient (*Apoe*^−/−^) or low-density lipoprotein receptor-deficient (*Ldlr*^−/−^) (115–118). While ER*α* non-nuclear signaling appears to play a critical role in protecting against vascular injury, ER*α* nuclear signaling is essential for preventing atherosclerosis by estrogen. This effect is preserved in C451A-ER*α* (98) and R264A-ER*α* mice (87), but abolished in ER*α*AF2^0^ mice (92) that are crossed with *Ldlr*^−/−^ mice. Notably, the AF1 domain of ER*α* appears to be dispensable for atherosclerosis prevention (91), suggesting the individual function of each domain.

### 4.3. Effect on metabolic homeostasis

Estrogen has been shown to suppress metabolic disorders, which are a common risk factor of CVDs. Female mice with whole-body ER*α* knockout exhibit several metabolic disorders, including glucose intolerance, body weight gain and visceral fat accumulation (96). Both nuclear and non-nuclear signaling of ER*α* appear to play a substantial role in these metabolic effects.

KRRKI mice also exhibits these dysfunctions, which are due to the disruption of the signal complex of membrane ER*α*, striatin, and PP2A in the hypothalamus, resulting in lower levels of physical activity and energy expenditure (104). In contrast, C451A-ER*α* mice only exhibit partial abnormalities (103), indicating that the mechanism of non-nuclear signaling inhibition may be relevant to the metabolic phenotype.

Regarding nuclear signaling, deletion of ER*α*AF2 induces similar abnormalities, with the disappearance of estrogen-regulated metabolic gene expression response to estrogen in the liver and adipose tissue. In contrast, deletion of ER*α*AF1 causes only mild hyperglycemia in the glucose tolerance test, suggesting a minor contribution of the AF1 domain (96).

### 4.4. Cardiac phenotype

E2 administration attenuates pressure overload-induced cardiac hypertrophy in female mice (119), as well as inhibits angiotensin II or endothelin-1-induced hypertrophy of neonatal rat cardiomyocytes (120). While ER*β* seems to play the predominant role in mediating estrogen's protective effect against hypertrophy (121–124), ER*α* also contributes to this effect (125).

The KRRKI mouse model has shed light on the role of ER*α* non-nuclear signaling in pressure overload-induced heart failure (89, 126). Phosphodiesterase 5 (PDE5) inhibitors prevent cardiac remodeling in mice by myocardial PKG activation (127). Interestingly, the efficacy of PDE5 inhibitors in female is dependent on estrogen, which stimulates cGMP synthesis *via* the eNOS/soluble guanylate cyclase pathway (128). Notably, in female KRRKI mice, PDE5 inhibitors failed to activate PKG and provide cardiac protection against pressure overload-induced heart failure, even in the presence of estrogen (89).

Other mouse models with genetic modification within the DBD (129, 130), LBD (131), and a transgenic mouse expressing only a functional E domain of ER*α* at the plasma membrane (132) also provide meaningful insights into intracellular estrogen signaling. However, the effect of these genetic modifications on the cardiovascular system have not been determined.

## 5. Selective stimulator of non-nuclear ER signaling

Estrogenic compounds and estrogen derivatives that selectively stimulate a subpopulation of ERs also offer important insights into non-nuclear signaling. Estradiol-bovine serum albumin conjugate (E2-BSA), estrogen-dendrimer conjugate (EDC), and pathway-preferential estrogens (PaPEs) have been developed as selective stimulator of non-nuclear signaling and widely used in various studies, including cardiovascular research.

### 5.1. Estradiol-bovine serum albumin conjugate

17*β*-estradiol conjugated to bovine serum albumin (E2-BSA) is membrane impermeable and therefore selectively stimulates cell-surface ERs. The exposure to E2-BSA leads to an increase in intracellular calcium and NO release in human artery endothelial cells, which is presumed to be mediated by non-nuclear signaling (133).

However, it should be noted that some criticisms have been raised regarding the suitability of E2-BSA as a tool to evaluate membrane-bound ER function: (1) E2-BSA solution contains some free E2 by cleaving from BSA, which may activate nuclear signaling; (2) the pattern of E2-BSA binding to ERs is influenced by the BSA linking site, which leads to different biological responses (134, 135).

### 5.2. Estrogen-dendrimer conjugate

A novel conjugate of estrogen and a polyamidoamine dendrimer was developed, in which the estrogens were linked to the dendrimer through a hydrolytically stable bond (136). This conjugate, known as estrogen-dendrimer conjugate (EDC), selectively stimulates ERs localized to the plasma membrane and cytoplasm since its positive charge and large size prevent it from entering the nucleus. EDC rapidly induces phosphorylation of ERK, Shc, and Src in MCF-7 cells, with limited impact on the expression of estrogen-responsive genes.

In bovine artery endothelial cells, EDC activates eNOS and promotes cell proliferation and migration. Moreover, EDC accelerates re-endothelialization of carotid artery after electric injury (137), which is consistent with the results of studies conducted on genetically modified mice.

In an ischemia/reperfusion model, E2 decreases cardiomyocyte apoptosis and infarct size (138, 139). Pretreatment with EDC similarly reduces infarct size and mitigates the decline in left ventricular function (140). This effect is accompanied by S-nitrosylation,of myocardial proteins, which plays an important role in cardioprotection (141).

It is noteworthy that continuous administration of EDC did not activate ER-mediated gene transcription *in vivo*, as evidenced by the bioluminescence assay using ERE-luciferase reporter mouse and real-time PCR (137). This finding not only confirms the high selectivity of EDC in stimulating non-nuclear signaling but also demonstrates the chemical stability of E2-dendrimer bound in EDC, which prevents the release of free E2.

### 5.3. Pathway-preferential estrogens

A novel estrogen compound that preferentially activates a subset of ERs has been developed through a distinct mechanism from EDC (142). Typically, an initial signal triggered by transient ER-ligand binding is sufficient to activate non-nuclear signaling, in which subsequent kinase cascades play a predominant role. In contrast, activation of nuclear signaling often requires sustained ER-ligand binding to induce a series of subcellular processes. Therefore, it is anticipated that the modified estrogen, which possesses appropriately reduced affinity to ERs, will activate non-nuclear signaling effectively while avoiding the stimulation of nuclear signaling.

Pathway-preferential estrogens (PaPEs) are synthesized from estradiol by altering its steroid structure and adding modifications. PaPE-1 binds to ER*α* and ER*β* 50,000 times less effectively than E2, while still retaining essential chemical features. PaPE-1 rapidly stimulates kinase phosphorylation in MCF-7 cells without directly activating genomic target genes. Additionally, PaPE-1 accelerated re-endothelialization of murine carotid arteries after electric injury (142).

In contrast to E2, neither PaPE-1 nor EDC prevents plaque formation in *Ldlr*^−/−^ female mice fed a hypercholesterolemic diet (98). This result confirms the pivotal role of ER nuclear signaling in the atheroprotective effect of E2, as demonstrated by ER*α*AF2^0^ mice (98).

## 6. Function of G-protein-coupled estrogen receptor (GPER)

GPER, called as GPR30 previously, constitutes a significant part of non-nuclear signaling of estrogen. GPER is a seven transmembrane G protein-coupled receptor (GPCR) of estrogen, identified in 1997 (143). GPER induces rapid activation of protein kinase A (PKA) as well as multiple signaling pathways that are also downstream of ER*α* (144–150).

Interestingly, GPER is not localized only at plasma membrane but also in intracellular compartments including endoplasmic reticulum and Golgi apparatus (151–99). The cellular distribution of GPER varies depending on the types of tissue and cells (151–154), and dynamically changes through intracellular trafficking (155, 156).

GPER mediates multiple physiological effects of estrogen on various organs, including the cardiovascular system. In contrast, it has been reported that GPER is dispensable for estrogenic effects in the reproductive system (94). Here, we outline the role of GPER in the cardiovascular system. For the detailed function and regulation of GPER, please refer to excellent comprehensive reviews elsewhere (157–159).

### 6.1. Vascular phenotype

G-1, a selective agonist of GPER (160), induces acute eNOS activation in rat aorta and cultured aortic endothelial cells (161). G-1 also activates protein kinase A in vascular smooth muscle cells, phosphorylating myosin light chain kinase (MLCK), while increasing intracellular calcium concentration, resulting in the relaxation of smooth muscle cells and thus vasodilatation (162–164). GPER-deletion in mice abrogates vasodilatory response to G-1 (105) and results in greater arterial constriction after vasoconstrictor exposure (165, 166) and development of high blood pressure with age (106).

GPER also mediates vasculo-protective effects of estrogen against atherosclerosis. In ovary-intact female mice fed on atherogenic diet, GPER deletion reduces vascular NO bioavailability and aggravates aortic inflammation and atherosclerosis (99), which contrasts with the dispensable role of ER*α* non-nuclear signaling in atherosclerosis (87, 98). G-1 induces differentiation of smooth muscle cell and suppresses proliferation (167), which could lead to the amelioration of atherosclerosis given the major pathogenic role of dedifferentiated smooth muscle cells (168).

### 6.2. Cardiac phenotype

GPER stimulation also confers cardiac protection in a PI3K-dependent mechanism. The pretreatment with G-1 attenuates contractile dysfunction and reduces infract size following ischemia/reperfusion injury (169), which is also accompanied by the suppression of proinflammatory cytokines in myocardium (170) and the inhibition of calcium-induced mitochondria permeability transition pore opening (171). Consistently, GPER-deficient male mice lose the protective effect of E2 against ischemia/reperfusion injury (100).

G-1 inhibits angiotensin II-induced cardiomyocyte hypertrophy (172). Studies using genetic models support the role of GPER in the heart. Over-expression of GPER using adeno-associated virus with G-1 stimulation ameliorates cardiac remodeling from chronic pressure-overload (173). Consistently, cardiomyocyte-specific GPER knockout induces cardiac dysfunction with increased cardiac oxidative stress and collagen deposition in female mice (101, 102).

### 6.3. Metabolic phenotype

GPER-deficient female mice lack estrogenic response to insulin, exhibiting hyperglycemia and glucose intolerance (106). Another line of GPER-null animals shows obese phenotype with visceral fat accumulation (105).

## 7. Conclusion

The series of studies focusing on non-nuclear signaling brought a new perspective on intracellular signaling and expanded our understanding of estrogen function. Further research advance would lead to a therapeutic approach that effectively distinguish cardiovascular protective effects from unfavorable ones such as cancer-progression and thrombosis.
